# Supplementary material for: Stationary-phase Pseudomonas aeruginosa fluoroquinolone persisters mostly avoid DNA double-stranded breaks
Source: mSphere. 2025 Dec 16;11(1):e00793-25. doi: 10.1128/msphere.00793-25 (PMC12838390; doi:10.1128/msphere.00793-25)
Supplement: Supplemental Material — s and methods, Tables S1–S3, Figures S1 and S2, supplemental video captions, and supplemental references. [file msphere.00793-25-s0001.pdf]

SUPPLEMENTAL MATERIALS

Stationary-Phase *Pseudomonas aeruginosa* Fluoroquinolone Persisters Mostly Avoid DNA Double-Stranded Breaks

Patricia J. Hare, Juliet R. González, Wendy W.K. Mok

Materials and Methods
Tables
    Table S1
    Table S2
    Table S3
Supplemental Figures
    Figure S1
    Figure S2
Supplemental Video Captions
    Video S1
    Video S2
References

Materials and Methods

Culture Media and Antibiotics

Cells were inoculated from frozen stocks into cation-adjusted Mueller-Hinton Broth (CA-MHB) and cultured for 4 h. CA-MHB was prepared from BD Difco Mueller Hinton Broth powder and cation-adjusted to final concentrations of 10 mg/L Mg^{2+} and 20 mg/L Ca^{2+}; cations were prepared as 85 g/L $MgCl_2•6H_2O$ and 28 g/L $CaCl_2$ stock solutions in water and filter-sterilized before adding to autoclaved MHB media. In order to decrease possible confounding factors due to batch variation of rich media, cultures for phenotypic assays were subcultured after the 4 h pre-growth into Basal Salt Media (BSM), a chemically defined minimal media with succinate as the sole carbon source (1, 2). BSM was prepared in water with 30.8 mM K_2HPO_4, 19.3 mM KH_2PO_4, 15 mM $(NH_4)_2SO_4$, 1 mM $MgCl_2$, 2 μM $FeSO_4$, and 15 mM succinic acid, then filter-sterilized before use. All liquid cultures were incubated at 37 ºC, shaking at 250 rpm.

Antibiotic stocks were prepared at the following concentrations: Levofloxacin (LVX) - 5 mg/mL in water, Tetracycline (Tet) – 10 mg/mL in water, Igrasan (Igr) - 25 mg/mL stock in ethanol, Carbenicillin (Carb) – 100 mg/mL in water, Ampicillin (Amp) – 100mg/mL in water. Agar plates were prepared as 1.5% agar. Antibiotics prepared in water were filter-sterilized using 0.22 μm polyethersulfone (PES) filters before use.

Preparation of the miniCTX-Gam-mScarlet Integrative Vector

P. aeruginosa Gam-mScarlet and mScarlet strains were generated using a miniCTX vector derivative (pJM252) which integrates genes of interest into the neutral *attB* site of the *P. aeruginosa* chromosome (3, 4). A gene fragment containing P_{lacIQ}-*lacI*-P_{tac}-mScarlet-I was ordered (Integrated DNA Technologies) with restriction sites on either end and amplified using lacIQ_Ptac_FWD2 and mScarlet_REV primers (see **Table S3**). The amplified gene was ligated into pJM252 by restriction digest at the KpnI and SacI cut sites. The ligation products were transformed into chemically competent *E. coli* DH5α and transformants were selected on Tet10 agar plates. Plasmids were confirmed by whole plasmid sequencing (Eurofins Scientific). The resultant miniCTX-Ptac-mScarlet vector was used as the backbone for creating the Gam-mScarlet translational fusion vector.

Gam was amplified from pRF3-Gam using primers Gam_FWD and Gam_REV, which contained restriction sites for SacI and SpeI, respectively (5). The Gam_REV primer also included an extra four alanine residues to create a flexible linker between Gam and mScarlet. The amplified gene was ligated into miniCTX-P_{tac}-mScarlet by restriction digest at the SacI and SpeI cut sites. The ligation products were transformed into chemically competent *E. coli* DH5α and cells were selected on Tet10 agar plates. Plasmids were confirmed by whole plasmid sequencing.

Cloning of Insertion Strains Using miniCTX Vector Derivatives

The *P. aeruginosa* parental strain ("recipient") was transformed via conjugation by triparental mating with *E. coli* containing the miniCTX derivative ("donor") and the helper strain *E. coli* HB101 pRK2013.

After triparental mating, transformed *P. aeruginosa* were selected for on LB agar containing Igr (25 µg/mL) and Tet (75 µg/mL). Three colonies on the LB-Igr25-Tet75 plate were grown in antibiotic-free LB for 2-3 h to cure miniCTX, then each clone was plated onto its own antibiotic-free LB agar plate to create a lawn. Simultaneously, lawns of *E. coli* SM10 + pFLP2 were plated onto LB + Carb (300 µg/mL) agar plates (6). The next day, pFLP2 was transformed into each *P. aeruginosa* mutant by biparental mating on LB agar. After 2 h mating, the mating spot was scraped, resuspended in LB, and dilutions were plated to LB agar and LB + 10% sucrose agar. The next day, sucrose-resistant clones were patched onto 1) LB agar, 2) LB + 10% sucrose agar, 3) LB + Carb300 agar, and 4) LB + Tet75 agar. Clones which were sucrose-resistant (indicates loss or inactivation of *sacB* gene on pFLP2), Tet-sensitive (indicates Tet resistance marker was cured from the miniCTX insert), and Carb-sensitive (indicates cured of pFLP2, a back-up to the 10% sucrose plate) were picked from the LB agar plate, grown in LB, and screened by cPCR for the insert of interest.

Whole Genome Sequencing

Genomic DNA was collected from individual clones grown in LB using the Qiagen Blood & Tissue Kit, according to the manufacturer's protocols. DNA was sent to SeqCenter (Pittsburgh, PA) for library preparation and sequencing. Libraries were prepared using the Illumina DNA Prep kit with custom 10 bp unique dual indices (UDI) with a target size of 280 bp. Libraries were sequenced on an Illumina NovaSeq X Plus (150 bp paired end reads, 2.67 million reads per sample). The resultant fastq read files were submitted to the online genome visualization tool, Proksee (7), for genome assembly (Proksee Assemble version 1.0.0a6) and feature annotation via Prokka (version 1.14.6) (8). To verify specific sequences, contigs with the regions of interest were downloaded from Proksee and compared to the desired sequences using SnapGene Viewer (version 7.0.1). Raw WGS reads can be found on the National Center for Biotechnology Information (NCBI) Sequence Read Archive under BioProject PRJNA1288492.

LVX Persistence Assays

P. aeruginosa strains were inoculated from -80 ºC frozen stocks into 2 mL test tubes of CA-MHB and grown at 37 ºC, shaking at 250 rpm. After 4-5 h of growth, inoculations were diluted 1:100 into 250-mL baffled flasks with 25 mL BSM + IPTG (1 mM). Following growth to stationary phase (16 h), OD_{600} of each culture was measured and 10 µL cells were collected for serial dilution in PBS and plating onto CA-MHB agar plates for colony forming unit (CFU) enumeration. Cells were then treated with 1 µg/mL LVX. At designated timepoints, 500 µL culture was collected and pelleted by centrifugation at 21,000 x *g* for 3 min. After removing 450 µL of supernatant, the pellets were washed with 450 µL PBS. This step was repeated, effectively diluting the antibiotics to subinhibitory levels (100-fold dilution). The cells were then serially diluted in PBS and 10 µL spots of each dilution were plated onto CA-MHB agar for CFU enumeration.

In order to test the LVX persistence of progenies derived from previously treated cells, 300 µL cells that were washed twice with PBS after 5 h treatment with LVX (1 µg/mL) or water (negative control) were stored in 25% glycerol stocks at -80 ºC overnight "for repeat treatment" (**Fig. S2A**). The following day, the frozen stocks were inoculated into 2 mL test tubes of CA-MHB, grown at 37 ºC, then diluted into pairs of 2 mL BSM test tubes each for growth to stationary phase (16 h).

Those tubes were then treated with LVX (1 μg/mL) or water and the persister assay was repeated as described above (**Fig. S2A**, "LVX survival assay with progeny").

Gam-mScarlet Assays and Time-Lapse Image Analysis of Gam Foci Formation

PAO1 and PA14 bearing the Gam-mScarlet translational fusion or the P_{tac}-*mScarlet* control were grown for 16 h to stationary phase in BSM + IPTG (1 mM). At t=0, the OD_{600} of each culture was measured and 10 μL cells were collected for serial dilution and plating onto CA-MHB agar for CFU enumeration. Then, the cultures were treated for 5 h with LVX (1 μg/mL). After treatment, cells were washed twice in PBS, and serially diluted for plating to CA-MHB for CFU enumeration. The washed cells were also diluted 30-fold in PBS for imaging. The cell dilutions were seeded onto antibiotic-free agarose pads made with BSM in a Bioptechs interchangeable cover dish (9). Cells were imaged every 10 min for 24 h recovery in a PeCon live cell incubation chamber kept at 37 ^{o}C.

Images were analyzed using Fiji (ImageJ2 version 2.9.0/1.53t) (10). Phase channel image stacks were merged with the OFP channel (for mScarlet-I fluorescence) then the merged stacks were drift-corrected using the Correct_3D_Drift.py script (11, 12).

Cell tracking and morphological/fluorescent signal classification were conducted with the MicrobeJ plugin (version 5.13l) (13). The scale was set to 0.1032 μm/pixel. In brief, *P. aeruginosa* bacterial cells were detected on the Phase channel with the following parameters: Area (0.4 -10 μm), Length (0.1 - 20 μm), Width (0-2.5 μm), Curvature (0-0.7), Angularity (0-0.2), Exclude on Edges, Shape Descriptors, and Segmentation. The Tracking option with Lineage analysis was selected.

From the Results table, 100 numbers were randomly selected from the total number of bacteria in the first frame (t=0) using a random number generator. Cells that were already dead at the first imaging timepoint were not included in the analysis, as their focal fluorescence status could not be determined. Cells that were dead at frame 0 were excluded from the list of 100 cells and replaced by the next eligible listed cell. Cells that were persisters were also replaced by the next eligible listed cell. The 100 non-persister cells were tracked and the frames in which they formed a fluorescent focus (fluorescent signal $\geq$ 2x fluorescence of the local area in the cell) and/or died (by explosive lysis or shrinking lysis and loss of phase contrast) were recorded. For persisters or cells that had not been LVX-treated, the frame in which they first divided was also recorded. Analyses were conducted on images from at least two independent experimental replicates.

UV Survival Assays

PAO1 and PA14 bearing the Gam-mScarlet translational fusion were grown for 16 h to stationary phase in BSM + IPTG (1 mM). At t=0, the OD_{600} of each culture was measured. 1 mL culture was diluted with 9 mL PBS and transferred into a 6.5 cm diameter Petri dish. Before UV exposure, 10 μL cells were collected for serial dilution and CFU enumeration. The lid of the Petri dish was removed and the UV lamp was turned on (254 nm, 4 Watt, 115 V, set 12 inches above the height of the Petri dish). At 30 sec, 1 min, and 5 min exposure time, 10 μL cells were collected for serial dilution and CFU enumeration. For the 0 and 5 min exposure samples, an additional 40 μL cells were collected and diluted with 80 μL PBS; 4 μL each sample was seeded onto BSM agarose pads in a Bioptechs interchangeable cover dish for imaging (9).

MIC Assays

MIC broth microdilution assays were conducted for progenies derived from untreated or LVX-treated cells according to CLSI standards (14). In brief, cells that were washed in PBS after treatment with/without LVX were resuspended for overnight recovery in CA-MHB ("liquid recovery") (**Fig. S2A**). Liquid recovery cultures were then diluted into test tubes with 2 mL CA-MHB to an OD_{600} 0.01-0.04 for growth to exponential phase. Additionally, cells that were derived from LVX-treated or untreated cells plated on LB agar ("plate recovery") were resuspended in PBS, then inoculated into 2 mL CA-MHB to an OD_{600} 0.01-0.04 for growth to exponential phase. At OD_{600} 0.2-0.4, cells were diluted to a concentration of 10^5 cells/mL in CA-MHB and 100 µL was added to each well of a 96-well plate already containing 100 µL 2x-concentrated drug in CA-MHB. The drug (LVX) was serially diluted two-fold for testing final concentrations between 0.03 and 16 µg/mL.

Statistical Analysis

All experiments were conducted over at least two biological replicates. Unless otherwise noted, statistical comparisons between samples were done by unpaired t-tests with Welch's correction for unequal variance at a significance level of 0.05.

Table S1. Bacterial strains used in this study.

Strain (Genotype)	Description	Source
P. aeruginosa **PAO1**	wild-type	Poole Lab, Queen's University (15)
**PAO1::P$_{tac}$-*gam-mScarlet***	IPTG-inducible Gam-mScarlet-I fusion	This work
**PAO1::P$_{tac}$-*mScarlet***	IPTG-inducible mScarlet-I	This work
P. aeruginosa **PA14**	wild-type NR-50573	BEI Resources
**PA14::P$_{tac}$-*gam-mScarlet***	IPTG-inducible Gam-mScarlet-I fusion	This work
E. coli **HB101 pRK2013**	F- *mcrB mrr hsdS*20(rB- mB-) *recA*13 *leuB*6 *ara*-14 *proA*2 *lacY*1 *galK*2 *xyl*-5 *mtl*-1 *rpsL*20(SmR) *glnV*44 λ- mobilization helper plasmid; Kan^R	Wu Orr Lab, Amherst College
E. coli **DH5α**	F- 80d*lacZ* M15 (*lacZYA-argF*) U169 *recA*1 *endA*1*hsdR*17(rk-, mk+) *phoA*supE44 -*thi*-1 *gyrA*96 *relA*1	Wu Orr Lab, Amherst College
E. coli **SM10 (λpir) pFLP2**	*thi thr leu tonA lacY supE recA*::RP4-2-TcR::Mu *KmR* λpir FLP recombinase plasmid; Carb^R	Wu Orr Lab, Amherst College

Table S2. Plasmids used in this study.

Plasmid (selective marker)	Description	Source
**pJM252** (Tet)	miniCTX-P_{tac}-P_{lacIQ}-*lacI* Integrative vector that localizes to the neutral *attB* site in the *P. aeruginosa* chromosome	Wu Orr Lab, Amherst College (3)
**pFLP2** (Carb)	FLPase-containing plasmid for curing FRT-flanked antibiotic resistance markers	Wu Orr Lab, Amherst College (6)
**pRF3-Gam** (Amp)	Vector with Gam-GFP	Rosenberg Lab, Baylor College of Medicine (5)
**miniCTX-mScarlet** (Tet)	Integrative vector with P_{lacIQ}-*lacI* and P_{tac}-*mScarlet-I*	This work
**miniCTX-gam-mScarlet** (Tet)	Integrative vector with P_{lacIQ}-*lacI* and P_{tac}-*gam-mScarlet-I*	This work

Table S3. PCR Primers used for this study.

Primer	Sequence (5' to 3')	Use
**lacIQ_Ptac_FWD2**	TCATCATGAGCTCGGATCCCGCTAACTTACATTAATTGCG	Amplify P_{lacIQ}-*lacI*-P_{tac}-mScarlet-I
**mScarlet_REV**	GTAGTAGGGTACCCTCGAGAAGCTTCTTGTACAGCTCGTC	Amplify P_{lacIQ}-*lacI*-P_{tac}-mScarlet-I
**Gam_FWD**	TCATCATGAGCTCCGTATCACGAGGCC	Amplify Gam from pRF3
**Gam_REV**	GTAGTAGACTAGTGGCGGCGGCGGCAATACCGGCTTCCTGTTCAA	Amplify Gam from pRF3

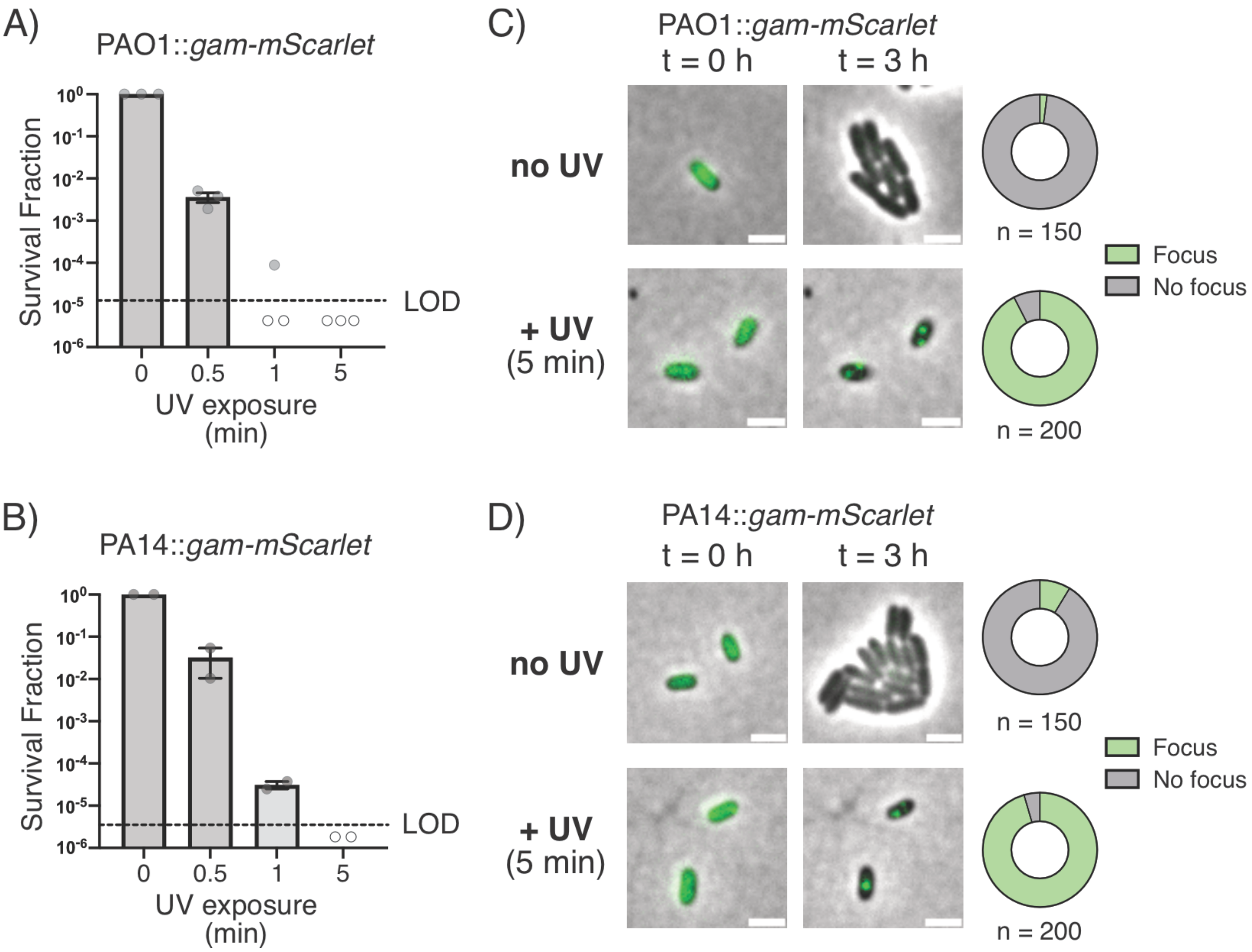

Figure S1. Gam-mScarlet forms foci on UV-induced DSBs in *P. aeruginosa*.
Survival fractions of **A)** PAO1::*gam-mScarlet* and **B)** PA14::*gam-mScarlet* after 0, 30 sec, 1 min, and 5 min UV exposure (n ≥ 2). LOD is the limit of detection for the assay, which was calculated based on the observed t = 0 colony counts for each strain. **C, D)** *P. aeruginosa*::*gam-mScarlet* cells exposed to UV light for 5 min were recovered on BSM agarose pads and observed to form foci indicative of DSBs (n = 200 cells per strain). Cells without UV exposure rarely formed foci (n = 150 cells per strain). The images shown are representative of two biological replicates. Scale bars represent 2 μm.

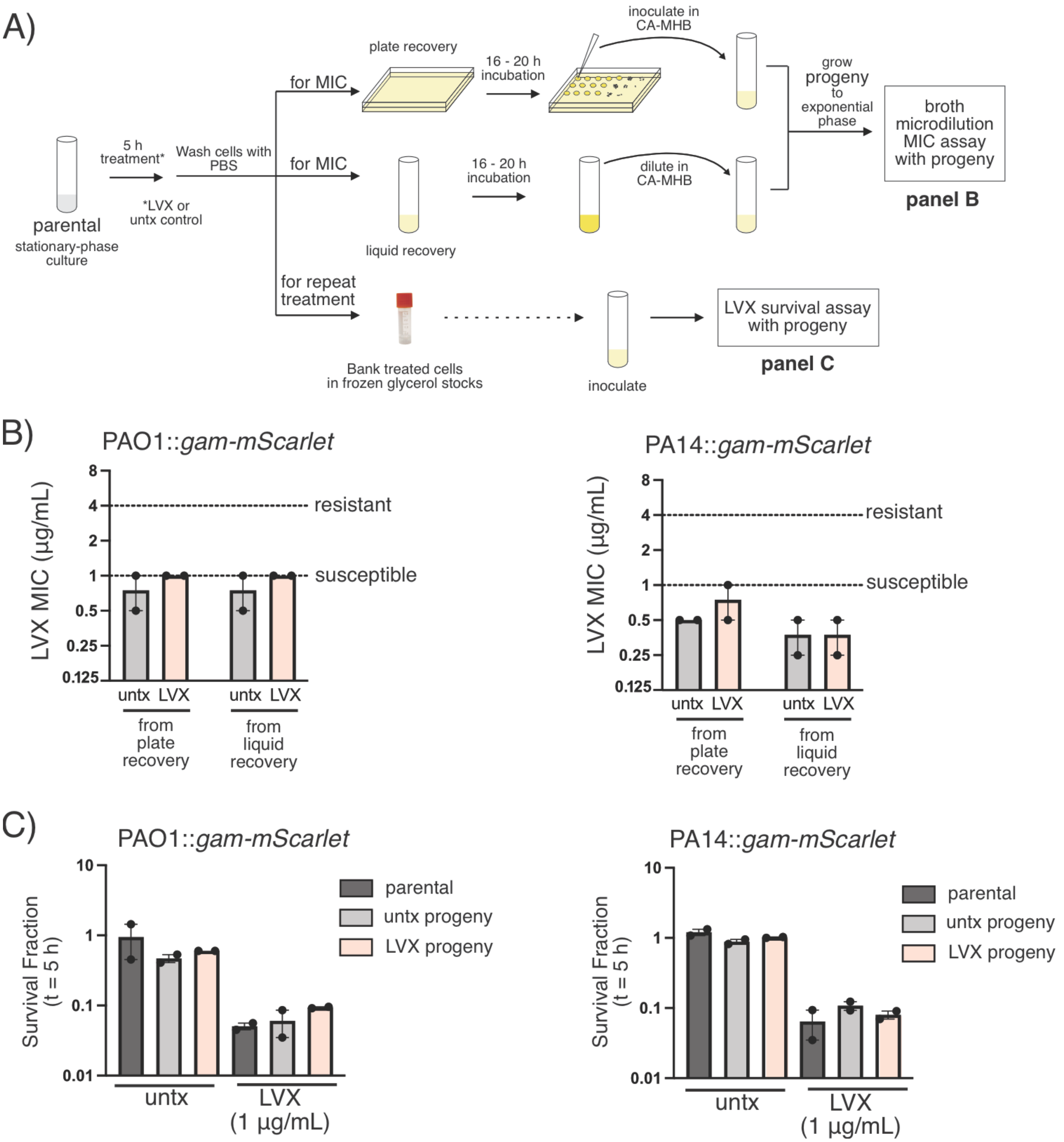

Figure S2. Cells that survive after 5 h LVX treatment are persisters, not LVX-resistant mutants.

A) Schematic of experimental protocols for testing whether the surviving LVX-treated cells are phenotypic variants (persisters) or the result of heritable genetic mutations that confer LVX resistance (n = 2). **B)** The minimum inhibitory concentrations (MICs) of persister progenies remain at or below the LVX susceptibility breakpoint whether cells were treated with LVX or untreated. **C)** Survival fractions after treatment with LVX (1 µg/mL) for 5 h are not increased for progenies of LVX persisters (n = 2).

<u>Supplemental Video Captions</u>

Video S1: Time-lapse fluorescence microscopy videos of *P. aeruginosa* PAO1::P$_{tac}$-*gam-mScarlet* persisters during recovery from LVX treatment (1 µg/mL).

These images are representative of three fields of view over two independent experiments. mScarlet fluorescence has been false-colored green for visual contrast. In each video, the cell of interest is centered and/or indicated by a box. The video shows the following persister phenotypes, in order: no fluorescent focus formation and gives rise to all healthy progeny, no fluorescent foci and progeny are split between healthy and non-dividing cells, focus partitions and progeny are split, focus resolves and progeny are split, focus resolves and progeny are all healthy. Images were taken every 10 min and the time of recovery is shown for each image as hh:mm.

Video S2: Time-lapse fluorescence microscopy videos of *P. aeruginosa* PA14::P$_{tac}$-*gam-mScarlet* during recovery from LVX treatment (1 µg/mL).

These images are representative of four fields of view over two independent experiments. mScarlet fluorescence has been false-colored green for visual contrast. In each video, the cell of interest is centered and/or indicated by a box. The video shows the following persister phenotypes, in order: no fluorescent focus formation and gives rise to all healthy progeny, no fluorescent foci and progeny are split between healthy and non-dividing cells, focus partitions and progeny are split, focus resolves and progeny are split, focus resolves and progeny are all healthy. Images were taken every 10 min and the time of recovery is shown for each image as hh:mm.

References

1. Wolff JA, MacGregor CH, Eisenberg RC, Phibbs P V. 1991. Isolation and characterization of catabolite repression control mutants of *Pseudomonas aeruginosa* PAO. J Bacteriol 173:4700-4706.
2. Robinson JL, Jaslove JM, Murawski AM, Fazen CH, Brynildsen MP. 2017. An integrated network analysis reveals that nitric oxide reductase prevents metabolic cycling of nitric oxide by *Pseudomonas aeruginosa*. Metab Eng 41:67–81.
3. Meisner J, Goldberg JB. 2016. The *Escherichia coli rhaSR-PrhaBAD* inducible promoter system allows tightly controlled gene expression over a wide range in *Pseudomonas aeruginosa*. Appl Environ Microbiol 82:6715-6727.
4. Hoang TT, Kutchma AJ, Becher A, Schweizer HP. 2000. Integration-proficient plasmids for *Pseudomonas aeruginosa*: Site-specific integration and use for engineering of reporter and expression strains. Plasmid 43:59-72.
5. Shee C, Cox BD, Gu F, Luengas EM, Joshi MC, Chiu L, Magnan D, Halliday JA, Frisch RL, Gibson JL, Nehring RB, Do HG, Hernandez M, Li L, Herman C, Hastings PJ, Bates D, Harris RS, Miller KM, Rosenberg SM. 2013. Engineered proteins detect spontaneous DNA breakage in human and bacterial cells. eLife 2:e01222.
6. Hoang TT, Karkhoff-Schweizer RR, Kutchma AJ, Schweizer HP. 1998. A broad-host-range Flp-*FRT* recombination system for site-specific excision of chromosomally-located DNA sequences: application for isolation of unmarked *Pseudomonas aeruginosa* mutants. Gene 212:77-86.
7. Grant JR, Enns E, Marinier E, Mandal A, Herman EK, Chen C, Graham M, Van Domselaar G, Stothard P. 2023. Proksee: In-depth characterization and visualization of bacterial genomes. Nucleic Acids Res 51:W484-W492.
8. Seemann T. 2014. Prokka: Rapid prokaryotic genome annotation. Bioinformatics 30:2068-2069.
9. Hare PJ, Batchelder JI, LaGree TJ, Mahey N, Power AD, Wu YI, Mok WWK. 2025. Time-lapse epifluorescence microscopy imaging of *Pseudomonas aeruginosa* and *Staphylococcus aureus* heterogeneous phenotypes. J Vis Exp 216:e67617.
10. Schindelin J, Arganda-Carreras I, Frise E, Kaynig V, Longair M, Pietzsch T, Preibisch S, Rueden C, Saalfeld S, Schmid B, Tinevez J, White DJ, Hartenstein V, Eliceiri K, Tomancak P, Cardona A. 2012. Fiji: An open-source platform for biological-image analysis. Nat Methods 9:676-82.
11. Parslow A, Cardona A, Bryson-Richardson RJ. 2014. Sample drift correction following 4D confocal time-lapse imaging. J Vis Exp 86:e51086.
12. Peng T, Thorn K, Schroeder T, Wang L, Theis FJ, Marr C, Navab N. 2017. A BaSiC tool for background and shading correction of optical microscopy images. Nat Commun 8:14836.
13. Ducret A, Quardokus EM, Brun Y V. 2016. MicrobeJ, a tool for high throughput bacterial cell detection and quantitative analysis. Nat Microbiol 1:16077.

14. Clinical and Laboratory Standards Institute. 2021. Performance standards for antimicrobial susceptibility testing. 31st ed, Wayne, PA.
15. Masuda N, Ohya S. 1992. Cross-resistance to meropenem, cephems, and quinolones in *Pseudomonas aeruginosa*. Antimicrob Agents Chemother 36:1847-1851.
